# Cell-Free DNA Variant Sequencing Using Plasma and AR-V7 Testing of Circulating Tumor Cells in Prostate Cancer Patients

**DOI:** 10.3390/cells10113223

**Published:** 2021-11-18

**Authors:** Verena Lieb, Amer Abdulrahman, Katrin Weigelt, Siegfried Hauch, Michael Gombert, Juan Guzman, Laura Bellut, Peter J. Goebell, Robert Stöhr, Arndt Hartmann, Bernd Wullich, Helge Taubert, Sven Wach

**Affiliations:** 1 Department of Urology and Pediatric Urology, Universitätsklinikum Erlangen, Friedrich-Alexander-Universität Erlangen-Nürnberg, 91054 Erlangen, Germany; Verena.Lieb@uk-erlangen.de (V.L.); Amer.Abdulrahman@uk-erlangen.de (A.A.); Katrin.Weigelt@uk-erlangen.de (K.W.); Juan.Guzman@uk-erlangen.de (J.G.); Laura.Bellut@uk-erlangen.de (L.B.); Peter.Goebell@uk-erlangen.de (P.J.G.); Bernd.Wullich@uk-erlangen.de (B.W.); sven.wach@uk-erlangen.de (S.W.); 2 Comprehensive Cancer Center Erlangen-EMN (CCC ER-EMN), 91054 Erlangen, Germany; Robert.Stoehr@uk-erlangen.de (R.S.); Arndt.Hartmann@uk-erlangen.de (A.H.); 3 QIAGEN GmbH, 40724 Hilden, Germany; Siegfried.Hauch@qiagen.com (S.H.); Michael.Gombert@qiagen.com (M.G.); 4 Institute of Pathology, University Hospital Erlangen, FAU Erlangen-Nürnberg, 91054 Erlangen, Germany

**Keywords:** prostate cancer, cfDNA, CTCs, prognosis, sequence variants

## Abstract

Prostate cancer (PCa) is the second most common malignant cancer and is a major cause of morbidity and mortality among men worldwide. There is still an urgent need for biomarkers applicable for diagnosis, prognosis, therapy prediction, or therapy monitoring in PCa. Liquid biopsies, including cell-free DNA (cfDNA) and circulating tumor cells (CTCs), are a valuable source for studying such biomarkers and are minimally invasive. In our study, we investigated the cfDNA of 34 progressive PCa patients, via targeted sequencing, for sequence variants and for the occurrence of CTCs, with a focus on androgen receptor splice variant 7 (AR-V7)-positive CTCs. The cfDNA content was associated with overall survival (OS; *p* = 0.014), disease-specific survival (DSS; *p* = 0.004), and time to treatment change (TTC; *p* = 0.001). Moreover, when considering all sequence variants grouped by their functional impact and allele frequency, a significant association with TTC (*p* = 0.017) was observed. When investigating only pathogenic or likely pathogenic gene variants, variants of the BRCA1 gene (*p* = 0.029) and the AR ligand-binding domain (*p* = 0.050) were associated with a shorter TTC. Likewise, the presence of CTCs was associated with a shorter TTC (*p* = 0.031). The presence of AR-V7-positive CTCs was associated with TTC (*p* < 0.001) in Kaplan–Meier analysis. Interestingly, all patients with AR-V7-positive CTCs also carried TP53 point mutations. Altogether, analysis of cfDNA and CTCs can provide complementary information that may support temporal and targeted treatment decisions and may elucidate the optimal choice within the variety of therapy options for advanced PCa patients.

## 1. Introduction

Prostate cancer (PCa) is a major cause of disease and mortality among men worldwide each year, and 1.4 million men are diagnosed with PCa. Approximately 375,000 men died in 2020 of PCa [1]. PCa is recognized as a genetically heterogeneous disease, comprising a large scope of malignancies, from indolent localized cancers that may never progress to rapidly progressing castration-resistant PCa (CRPC) [2]. In accordance with the recent knowledge of clinicopathologies and genetics, the World Health Organization’s (WHO) classification of prostatic cancers has been revised, and five grade groups have been established [3]. However, this classification is based on biopsy or prostatectomy specimens. A valuable source of information is a noninvasive liquid biopsy, which can be used to characterize different components of body fluids, including circulating tumor cells (CTCs), cell-free DNA (cfDNA), circulating RNA, microRNAs, and extracellular vesicles (EVs), and can be prognostic of PCa outcome, predictive of response to treatment, or used to monitor disease [4].

CTCs in PCa have been suggested as potential surrogate markers of survival [5]. Numerous studies have identified a relationship between CTC counts and disease progression or overall survival in metastatic castration-resistant prostate cancer (mCRPC) [6,7,8,9]. Recently, a prognostic impact of CTC enumeration has also been shown for hormone-naïve oligometastatic PCa and localized PCa patients [10,11]. CTCs can mirror the bone metastatic phenotype in PCa [12]. There are several methods for detection and characterization of CTCs, reviewed in [4], among which the CellSearch system is still the only one approved by the FDA; additionally, RNA-based RT–PCR tests, such as AdnaTest, have been applied for different tumor entities, including PCa [13,14,15,16]. The presence of CTCs detected with the AdnaTest is a negative prognostic marker for progression-free survival [17]. For PCa, detection of androgen receptor splice variant 7 (AR-V7) in CTCs is associated with shorter progression-free, overall, and tumor-specific survival than that in patients with AR-V7-negative CTCs [15,16,18,19]. In addition, mCRPC patients with AR-V7-positive CTCs detected at the RNA or protein (nucleus) level has been shown to respond better to taxanes than to AR-inhibitory therapies [20,21,22]; however, a small group of CRPC patients with AR-V7-positive CTCs can still respond to AR-inhibitory therapies [23]. Therefore, further characterization of AR-V7-positive CTC-carrying patients is necessary to stratify them for individualized therapy.

CfDNA and the included circulating tumor (ct) DNA have been studied in plasma samples of patients with different tumor entities, and the load of cfDNA might be correlated with tumor staging and prognosis [24]. In PCa, the amount of cfDNA has diagnostic potential, i.e., it is on average higher in PCa patients than in BPH patients or control probands [4,25,26,27,28,29]. In addition, high cfDNA values were significantly associated with pathologic T3 stage [26]. High cfDNA or high ctDNA fractions (or both) are associated with poor prognosis in PCa patients [27,29,30,31]. Furthermore, they might be correlated with indices of overall tumor burden in mCRPC patients [30]. There is consensus that ctDNA has a high overlap with genomic sequences of metastatic biopsies and is representative of multiple metastases [30,32]. In CRPC, several genomic alterations have been identified in ctDNA, such as in the androgen receptor, whose alteration is associated with the outcome of anti-androgen therapies, as well as in DNA repair genes associated with the response to PARP inhibitors ([33,34] reviewed in [32]). However, androgen deprivation therapy (ADT) rapidly reduces ctDNA availability [35], which complicates its characterization and potentially makes the pure quantification of cfDNA as a prognostic marker in PCa patients questionable.

Overall, whether and how much the information gained from CTCs and from cf/ctDNA partially overlaps or is complementary is still a matter of debate. Whereas CTCs are living cells that originate from primary or metastatic cancers, ctDNA stems from tumor cells of the primary tumor, metastases, or CTCs dying via apoptosis or necrosis or that are therapy sensitive [14,32]. Interestingly, cfDNA reflects metastasis, rather than the primary tumor; thus, studying cfDNA may reduce the need for metastatic biopsies [36]. In this study, on the one hand, we were interested in analyzing the number of CTCs and their AR-V7 expression and, on the other hand, we were interested in quantifying cfDNA and characterizing its genomic alterations in the same metastatic PCa patients.

## 2. Materials and Methods

### 2.1. Patients and Tumor Material

In this study, 39 blood samples from 34 PCa patients, collected in the University Hospital Erlangen from 2016–2019, were included. The research on human subjects was performed in compliance with the Helsinki Declaration. All patients provided written informed consent. The study was approved by the Ethics Commission of the University Hospital Erlangen (No. 3755 and No. 329_16B). Tumor histology was reviewed by an experienced uropathologist (AH). An overview of the clinicopathological data, treatment data, and study data of the patients is provided in Table 1.

### 2.2. Sampling of Blood, Isolation of CTCs, and Processing of Plasma

Blood samples were collected via venipuncture in K_3_EDTA anti-coagulation tubes (Sarstedt AG & Co KG, Nümbrecht, Germany), stored at 4 °C, and processed within 4 h after the blood was drawn.

Plasma was prepared by centrifugation at 2000× *g* for 10 min and stored as aliquots at −80 °C. Cell-free DNA was isolated from 4–8 mL plasma via affinity-based binding to magnetic beads (QIAamp MinElute ccfDNA Kit, QIAGEN, Hilden, Germany), according to the manufacturer’s instructions, and was quantified via fluorometry, using a Quantus fluorometer (Promega, Maddison, WI, USA). Plasma cfDNA concentrations were normalized to the plasma input volume and are given in ng/mL.

### 2.3. Library Construction and Sequencing

The breast cancer panel (DHS-001Z-96; Qiagen, Hilden, Germany), comprising 93 genes, was boosted by 6 additional PCa-relevant genes (TMPRSS2, ERG, ERCC1, ERCC3, FOXA1, and SPOP). From 30 PCa patients, 1 sample was analyzed; from 3 patients, 2 samples were analyzed; and from 1 patient, 3 samples were analyzed.

Library preparation was performed using a QIAseq Targeted DNA Panel Library Prep Kit. An amount of 10 ng, or the maximum when 10 ng of starting material was not available, was enzymatically fragmented. Ends were repaired and 3′ adenylated. Adapters were ligated to the overhang, and the reactions were cleaned up. Target regions were enriched via target-specific PCR (22 cycles). After bead-based reaction cleanup, molecules were enriched via 22 cycles of universal PCR, and a final cleanup was performed. Library preparation was quality controlled using capillary electrophoresis (Agilent DNA 7500 Chip). High-quality libraries were pooled, based on equimolar concentrations using a bioanalyzer automated electrophoresis system (Agilent Technologies). The library pool(s) were quantified using qPCR, and the optimal concentration of the library pool was used to generate the clusters on the surface of a flow cell before sequencing on a NextSeq (Illumina Inc., San Diego, CA, USA) instrument (2 × 151 bp, 2 × 8 bp), according to the manufacturer’s instructions (Illumina Inc., San Diego, CA, USA).

### 2.4. Data Analysis and Bioinformatics Analysis

Primary bioinformatic analysis was carried out with the CLC Genomic Workbench. Read quality control was facilitated with the “QC for Sequencing Reads” tool. Adapters were trimmed by the “Trim Reads” tool, and alignment, UMI grouping, and variant calling were facilitated by the “Identify QIAseq DNA Somatic Variants” workflow.

Finalized variant files were introduced to the Qiagen Gene Globe data analysis application. By using the QCI translational application (QIAGEN Clinical Insight Interpret 8.0.20210827, Hilden, Germany) with precurated databases for somatic variations, the results were further refined. Briefly, all common SNPs with a known allele frequency above 1% were omitted. Furthermore, any sequence variations located within intronic sequences more than 20 bp from the exon–intron border were also omitted. Additionally, sequence variants with a known pathogenic function were explicitly included in the analysis. According to the QCI translational precured databases, all sequence variants were classified by their biological function (loss of function, gain of function, or normal function), as well as their pathological impact (pathogenic, likely pathogenic, benign, likely benign, or uncertain impact). The final variation set included a total of 2505 sequence variations distributed over 98 genes.

### 2.5. Isolation of mRNA and qRT–PCR

CTCs were isolated from 5 mL of whole blood via positive immunomagnetic selection by applying the AdnaGen prostate cancer select system (QIAGEN GmbH, Hilden, Germany), targeting EpCAM and PSMA. Cellular mRNAs were isolated via immunomagnetic enrichment (oligo dT(25)-coated magnetic beads) and reverse transcribed. The mRNAs of CD45, PSA, PSMA, AR, AR-V7, and GAPDH were quantified using the AdnaTest ProstateCancerPanel AR-V7 (Qiagen, Hilden, Germany). PSMA and GAPDH detection were considered for verification of CTCs, and additional detection of AR-V7 was considered for verification of AR-V7-positive CTCs. Exclusive detection of CD45 or GAPDH (or both) was considered as verification of lymphocytes.

### 2.6. Statistical Analysis

Correlations between the detection of cfDNA, CTCs, and sequencing results and clinicopathological data were calculated using Spearman’s correlation test, a chi-squared test, or a Mann–Whitney test. The associations of cfDNA, CTCs, AR-V7-positive CTCs, and sequencing results with overall survival (OS), disease-specific survival (DSS), and time to treatment change (TTC) were determined by univariate analyses (Kaplan–Meier analysis with log-rank test and Cox’s regression hazard models). A *p* value less than 0.05 was considered statistically significant. Statistical analyses were performed using the SPSS 21.0 software package (SPSS Inc., Chicago, IL, USA). For hierarchical clustering, the Euclidean distance and average linkage were used, and the analysis was performed using R statistical framework Ver. 3.2.1 (R Foundation for Statistical Computing, Vienna, Austria. http://www.R-project.org/ (accessed on 20 October 2021)).

## 3. Results

**Figure 1:**
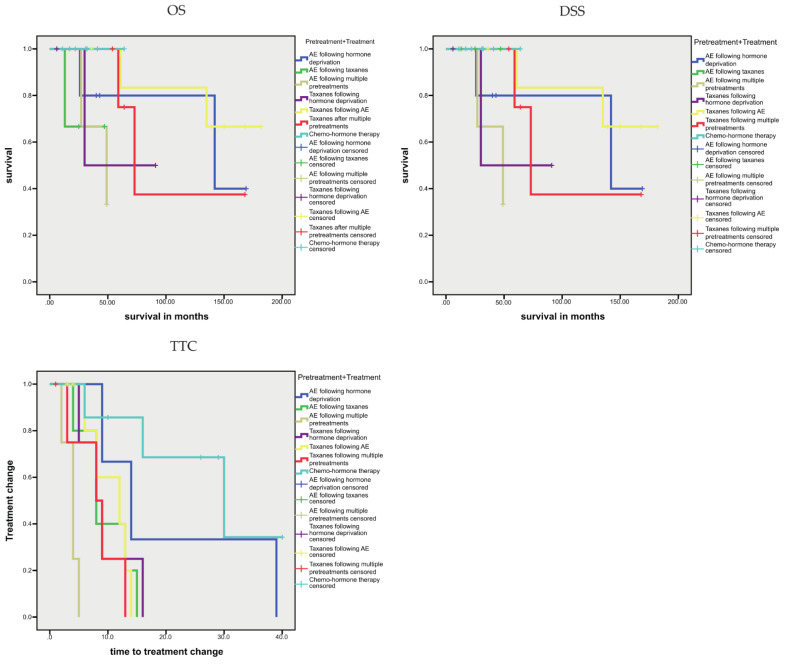
Kaplan–Meier analysis: association of pretreatment–treatment groups with prognosis (OS, DSS, TTC).

We studied 39 peripheral blood samples from 34 PCa patients with progressive PCa for plasma cfDNA sequence variants. Out of these for 37 samples (33 patients), the presence of CTCs was analyzed with a focus on CTCs expressing the androgen splice variant AR-V7 (AR-V7-positive CTCs). An overview of the clinicopathological data, treatment schemes, and study data is given in Table 1. The patients were grouped according to their pretreatment into five groups, as follows: no pretreatment; basic hormone deprivation (e.g., leuprorelin); antiandrogens abiraterone/enzalutamide (AE); taxanes; or multiple (both AE and taxanes). In addition, the treatment (performed subsequent to blood sampling and prior to therapy change) consisted of three schemes: AE, taxanes, or chemohormone therapy. According to pretreatment and treatment, we separated the patients into seven groups, as follows: chemohormone therapy without pretreatment; AE following hormone deprivation; AE following taxanes; AE following multiple pretreatments; taxanes following hormone deprivation; taxanes following AE; and taxanes following multiple pretreatments. The first clinical end point was the time to treatment change (TTC), which was defined as the interval between blood sampling (including the scheduled therapy scheme) and the initiation of the following therapy scheme. Other clinical endpoints, including the interval between diagnosis and the last date of patient information, were obtained to define overall survival (OS) and disease-specific survival (DSS). In the Kaplan–Meier analysis, we studied the association between these pretreatment–treatment groups and prognosis, i.e., OS, DSS, and TTC (Figure 1).

Concerning the underlying prognostic characteristics, we discovered in a pairwise comparison that patients treated with AE following multiple pretreatments had poorer survival (both OS and DSS) than patients receiving taxanes following multiple pretreatments (both *p* = 0.043) or patients receiving taxanes following AE (both *p* = 0.017; Figure 1).

Kaplan–Meier analysis of the association between the pretreatment–treatment groups and TTC was performed with the chemohormone therapy group as the reference. Here, all groups, with the exception of patients receiving AE following hormone deprivation, showed a significantly shorter TTC, i.e., AE following taxanes (*p* = 0.034), taxanes following hormone deprivation (*p* = 0.004), taxanes following AE (*p* = 0.019) and taxanes following multiple pretreatments (*p* = 0.028; Figure 1).

Next, we were interested in whether a liquid biopsy—i.e., analysis of cfDNA, CTCs, and AR-V7-positive CTCs—could provide further information concerning molecular changes and the prognosis of PCa patients.

### 3.1. Analysis of Plasma-Derived cfDNA

#### 3.1.1. Correlation of cfDNA Concentration with Clinicopathological and Molecular Data

The concentration of cfDNA was in the range of 2.37 ng/mL–206.25 ng/mL (mean 17.93 ng/mL; median 10.56 ng/mL; Table 2). The level of cfDNA was correlated with the follow-up time after blood sampling (r_s_ = −0.333; *p* = 0.047) and the presence of AR-V7-positive CTCs (r_s_ = 0.346; *p* = 0.036) tended to be correlated with the TTC (r_s_ = −0.304; *p* = 0.071; Table 3). However, the concentration of cfDNA was not correlated with age, PSA level, or the pretreatment scheme (data not shown).

**Figure 2:**
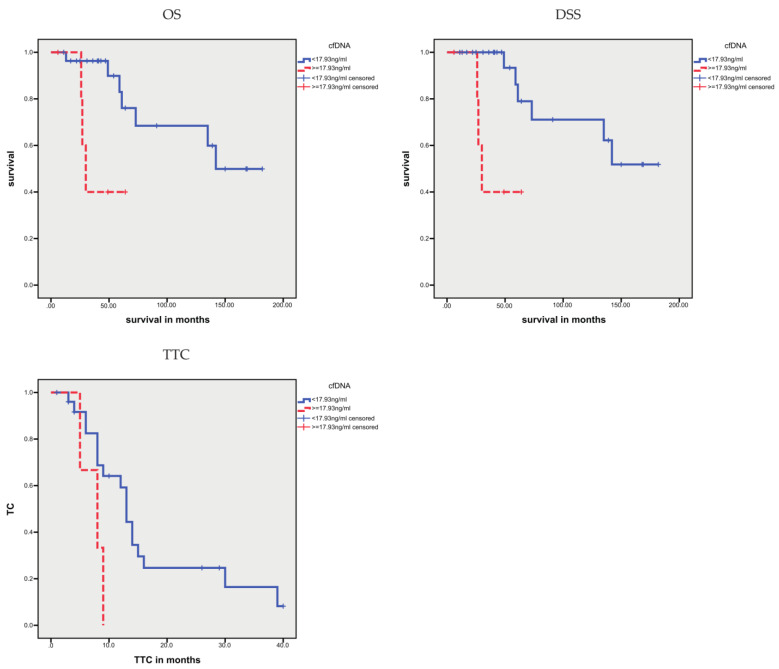
Kaplan–Meier analysis: association between cfDNA concentration and prognosis (OS, DSS, and TTC). A higher cfDNA concentration (> 17.93 ng/mL) was associated with a shorter OS (*p* = 0.014), DSS (*p* = 0.004), and TTC (*p* = 0.001).

Next, we stratified patients, according to the mean of their cfDNA concentration, into two groups (≤ 17.93 ng/mL vs. > 17.93 ng/mL). Prognosis was analyzed via Kaplan–Meier analysis (Figure 1). A higher level of cfDNA was associated with a shorter OS (*p* = 0.014; 42.2 vs. 134.0 months) and a shorter DSS (*p* = 0.004; 42.2 vs. 138.7 months; Table 4). Next, we were interested in whether the level of cfDNA was associated with the TTC. A higher level of cfDNA was associated with a significantly shorter TTC (*p* = 0.001; 6.3 vs. 16.2 months) than that observed in patients with a lower level of cfDNA (Figure 2; Table 4). In univariate Cox’s regression analysis, the patients with higher cfDNA levels had a 5.53-fold increased risk of death (*p* = 0.027) and a 7.87-fold risk of disease-specific death (*p* = 0.013; Table 5), compared with patients with lower levels of cfDNA. Likewise, the risk for an earlier treatment change in the group with higher cfDNA levels was 4.11-fold increased (*p* = 0.004) compared with the group with lower levels of cfDNA (Table 5).

#### 3.1.2. Sequence Variants Detected in cfDNA

Targeted sequencing of cfDNA was performed for a total of 99 genes. After applying the data filtering schemes described in the methods section, a total of 2505 sequence variations were identified in our patient cohort. Of these, the classification was as follows: 1835 uncertain variants, 164 likely benign variants, 31 benign variants, 387 likely pathogenic variants, and 61 pathogenic variants (Table S1; Figure S1). The occurrence of individual sequence variations in the 39 samples differed from a single detection in one sample for 2049 variants, two independent detections for 129 variants, and three independent detections for 54 variants. A total of 273 variants were detected in four or more of the samples. Considering the pathological impact—i.e., only the likely pathogenic and pathogenic variants—448 variants distributed on 68 genes were identified. At a frequency of ≥2; 375 variants were found distributed on 66 genes. By combining the pathological impact (likely pathogenic or pathogenic) and the frequency of occurrence, 64 variants distributed on 22 genes were detected (Table S1). The variants comprised SNVs, insertions or deletions of variable length, and substitutions of ≥2 base pairs. Interestingly, most of the variants resulted in a loss of function and were related to genes considered tumor suppressors or DNA repair genes, or both. Taken together, all samples analyzed likely harbored pathogenic and pathogenic variants.

Considering the patient with three sequential samples analyzed, it was of interest that the number of detected variants decreased with tumor progression. However, pathogenic variants that occurred in the first sample were retained in the second and third samples, e.g., in the PTEN gene (c.916G > A and c.926G > A), in the CHEK2 gene (c.1556C > T), and in the TP53 gene (c.857A > C).

#### 3.1.3. Frequency of Variants

Of the pathogenic and likely pathogenic variants (n = 424), a total of 56 (13.2%) were located in the MUC16 gene and 33 variants (7.8%) were located in SYNE1 (spectrin repeat-containing nuclear envelope; synonymous: Nesprin1), and with 32 variants (7.5%), the AR gene contained the third highest variant frequency. However, when we normalized the frequency of pathogenic and likely pathogenic variants per covered sequence (in kb), the TP53 gene, with a ratio of 10.85 (15 variants/1.383 kb), showed the highest ratio; the AR gene, with a ratio of 10.65 (32 variants/3.005 kb), exhibited the second highest ratio; and the CHEK2 gene, with a ratio of 7.85 (15 variants/1.911 kb), exhibited the third highest ratio (Figure S2); whereas, MUC16, with a ratio of 1.26, and SYNE1, with a ratio of 1.18, presented rather moderate ratios (Table S1).

#### 3.1.4. Association between Sequence Variants and Prognosis

**Figure 3:**
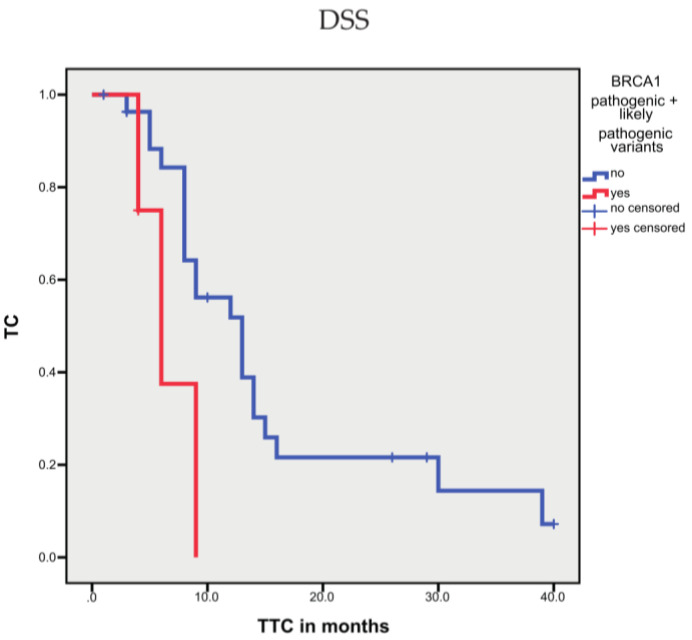
Kaplan–Meier analysis: association between BRCA1 sequence variants and time to treatment change (TTC). Patients with BRCA1 likely pathogenic and pathogenic sequence variants showed a shorter TTC (*p* = 0.029).

**Figure 4:**
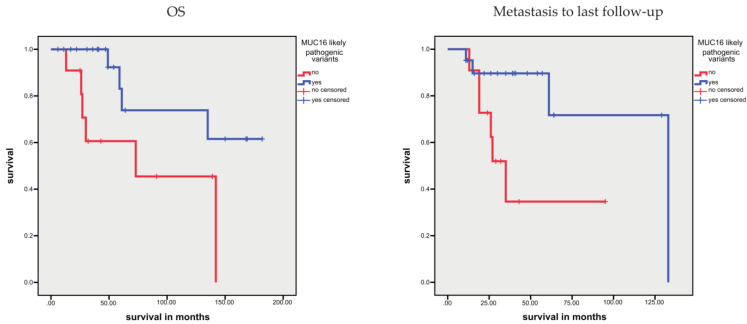
Kaplan–Meier analysis: association between MUC16 likely pathogenic sequence variants and OS or time of metastasis to last follow-up. Patients with likely pathogenic variants in the MUC16 gene showed a longer OS (*p* = 0.028) and a longer survival time after diagnosis of metastasis (*p* = 0.025).

Considering only pathogenic and likely pathogenic variants for single genes, several genes, such as TP53, AR, RAD51, and RB, did not show an association with prognosis (OS, DSS, and TTC; data not shown). However, patients with BRCA1 pathogenic and likely pathogenic variants, but not those with BRCA2 variants, showed a shorter TTC (*p* = 0.029; Figure 3); whereas, OS or DSS were not associated with pathogenic or likely pathogenic variants in either gene. Keup et al. reported that three variants in the MUC16 gene in metastatic breast cancer were associated with a longer survival time after diagnosis of metastasis [37]. Interestingly, in our study, patients with likely pathogenic variants for the MUC16 gene showed a longer OS and a longer survival time after diagnosis of metastasis (*p* = 0.028 and *p* = 0.025; Figure 4). We also tested the large gene SYNE1, but pathogenic and likely pathogenic variants were not associated with prognosis.

**Figure 5:**
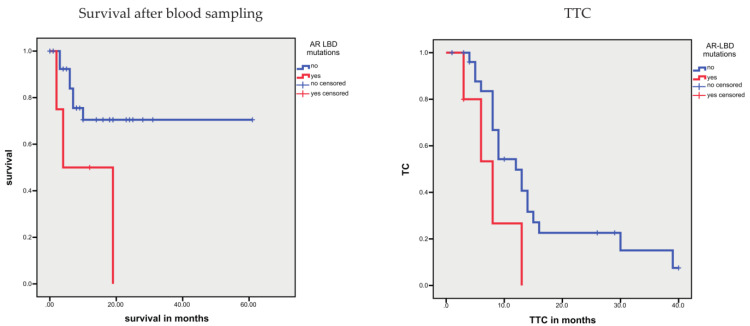
Kaplan–Meier analysis: AR-LBD mutations are associated with survival after blood sampling and TTC. Patients with AR-LBD mutations showed a shorter survival after blood sampling (*p* = 0.036) and, as a trend, a shorter TTC (*p* = 0.050).

Through a more detailed analysis, Conteduca et al. found that two AR gene mutations in the ligand binding domain (LBD; 2105T > A (p. L702H) and 2632A > G (p. T878A)) were associated with a shorter OS in PCa [33]. We detected these two AR point mutations in four patients, and two of these patients possessed both mutations simultaneously. In addition, two patients showed other AR-LBD gene variants (Table S1). In our study, for all patients with AR-LBD mutations, an association with a shorter survival time after blood sampling (*p* = 0.036) and a trend toward a shorter TTC were found (*p* = 0.050; Figure 5), when compared with patients without mutations in the AR-LBD.

**Figure 6:**
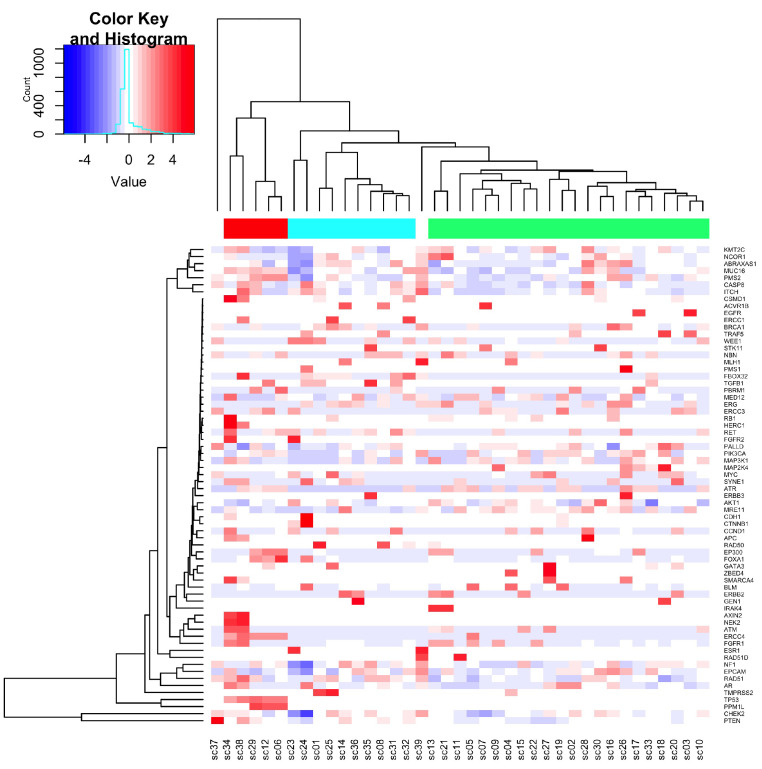
Heatmap variant score. Hierarchical clustering revealed three distinct clusters. The variant score of individual genes (right hand side) and for all individual samples (bottom lane) was clustered using Euclidian distance and average linkage. Hierarchical clustering revealed three distinct clusters marked by the color bars on the top with Group 1 in light blue, Group 2 in green and Group 3 in red.

**Figure 7:**
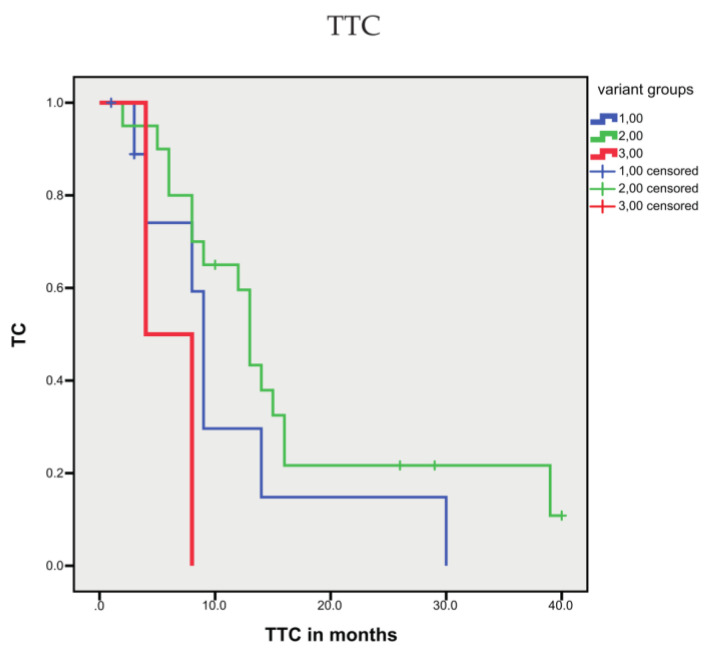
Association of sequence variant groups with TTC. The three sequence variant groups showed a significant association with TTC (*p* = 0.017).

To assess both the functional impact of detected variants and the frequency of variant alleles, we calculated a gene wise and sample variation score. Hereby, the inferred functional impact of the variant was rated as 0 (variant not present), 1 (normal function), or 2 (loss of function or gain of function). These ratings were multiplied by the allele frequency of the variant allele. To calculate a gene wise, size-normalized score, the variation values were summarized for the observed gene and divided by the sequence length assessed by targeted sequencing. Hierarchical clustering revealed three distinct clusters (Figure 6). The three clusters only tended to be correlated with OS (r_s_ = −0.300; *p* = 0.095) or DSS (r_s_ = −0.345; *p* = 0.053; Table 3). However, the three clusters showed a significant association with TTC (*p* = 0.017; Figure 7).

### 3.2. Analysis of CTCs

#### 3.2.1. Isolation of CTCs and Correlation with Clinicopathological and Molecular Data

Out of the 39 samples, 37 samples were also available to assess the presence of CTCs and the expression of AR-V7. In 11 samples, we detected CTCs, and in 4 of these samples, AR-V7-positive CTCs were found (Table S2). The presence of CTCs was correlated with the variant groups (r_s_ = 0.472; *p* = 0.004; Table 3). The presence of AR-V7-positive CTCs was correlated with the time of blood sampling to follow-up (r_s_ = −0.377; *p* = 0.028) and TTC (r_s_ = −0.378; *p* = 0.028). However, neither the presence of CTCs nor that of AR-V7-positive CTCs was correlated with AR-LBD mutations (data not shown).

TP53 gene alterations are the most commonly occurring gene alterations in PCa [38]. TP53 alterations can be differently associated with prognosis in cancer [39]. Accordingly, we separated our patients into 3 groups according to their pathogenic and mostly pathogenic TP53 gene variants: (Group 1) without gene variants, (Group 2) with frameshift mutations, and (Group 3) with point mutations. The 3 TP53 variant groups were associated with AR-V7-positive CTCs (r_s_ = 0.523; *p* = 0.001; Table 3) and trended to be associated with the occurrence of CTCs (r_s_ = 0.309; *p* = 0.063). Interestingly, CTCs occurred predominantly in the 2 groups with TP53 sequence variants (7/11) and in 5 cases in Group 3 (with TP53 point mutations; Table S3). All 4 samples with AR-V7-positive CTCs were found in Group 3 (*p* < 0.001; Table S3).

#### 3.2.2. Associations between CTCs and Prognosis

**Figure 8:**
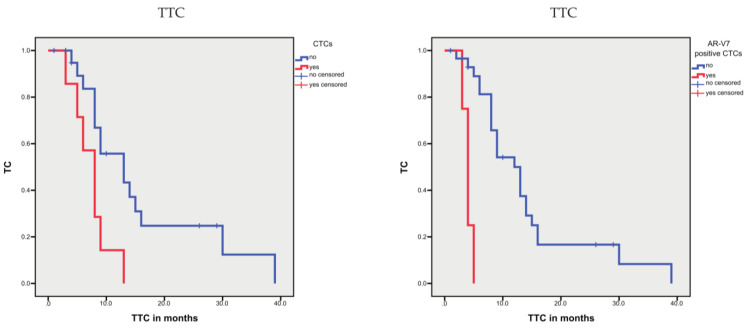
Kaplan–Meier analysis: association of CTCs or AR-V7-positive CTCs with time to treatment change (TTC). The presence of CTCs or AR-V7-positive CTCs was associated with a shorter TTC (*p* = 0.031 or *p* < 0.001).

Based on Kaplan–Meier analysis, the presence of CTCs was not associated with OS or DSS but was associated with a shorter TTC (*p* = 0.031; 7.6 vs. 15.1 months; Table 4; Figure 8) compared with patients with no CTCs. In univariate Cox’s regression analysis, patients with CTCs possessed a 2.32-fold increased risk for a shorter TTC (*p* = 0.046; Table 5) compared with patients without CTCs.

The presence of AR-V7-positive CTCs was also not associated with OS but showed an association trend with DSS (*p* = 0.097; 57.7 vs. 134.8 months) and was significantly associated with TTC (*p* < 0.001; 4.0 vs. 14.2 months; Table 4; Figure 8); however, it must be considered that only 4 samples from 3 patients showed AR-V7-positive CTCs. In the univariate Cox’s regression analysis, the presence of AR-V7-positive CTCs was associated with a 14.75-fold increased risk of treatment change (*p* = 0.001) but not with an increased risk of disease-specific death (Table 5).

## 4. Discussion

We studied a group of PCa patients with progressive disease in different pretreatment and treatment stages. As expected, the chemohormone therapy group, being in the beginning of therapy, showed the longest OS, DSS, and TTC. In contrast, but equally expected, the AE group following multiple pretreatments presented with the shortest OS, DSS, and TTC. However, we were interested in whether molecular characterization of cfDNA and CTCs could provide insight into associations with the prognosis of these PCa patients. We performed targeted cfDNA sequencing for sequence variant identification and AR-V7 testing of CTCs from the patients.

Quantitative assessment of cfDNA can be used as a diagnostic biomarker and to assess tumor burden and response to therapy [40]. In our study, the amount of cfDNA found (approximately 18 ng/mL plasma) was in the range found previously in many other cancer types, including PCa (average 18 ng/mL to 31.9 ng/mL) [41,42]. However, the level was somewhat below what is described on average for CRPC [41]; however, it is well known that the amount of cfDNA is prone to change during treatment [43]. The concentration of cfDNA was associated with the prognosis of PCa patients. A higher cfDNA concentration—i.e., above the mean value (17.93 ng/mL)—was associated with a shorter OS and DSS, which is in accordance with previous findings of other authors [27,29,31,34,43,44,45]. In addition, higher levels of cfDNA were associated with a shorter TTC. However, our cfDNA concentrations are not baseline values and therefore mainly reflect therapy response. Several studies report a reduction in cfDNA concentrations between baseline and after therapy for responders, e.g., after chemotherapy [44] or PARP inhibition [34].

However, in addition to the amount of cfDNA, the mutational spectrum has relevance for prognosis and therapy decisions [46,47,48]. Considering only the pathogenic and likely pathogenic variants, all patients showed such variants in their cfDNA, and as expected, the TP53 gene and AR gene exhibited these variants most frequently in our study. This finding is not unexpected; Robinson et al. showed that mutations in the TP53 and AR genes are the most frequent in mCRPC [38], and cf/ctDNA has been demonstrated to be representative of multiple metastases [30,32]. Recently, TP53 status in cfDNA of CRPC patients has been reported as an independent prognostic factor for progression-free survival [49]. However, TP53 mutations can be an early event in some PCa, while in others, an enrichment in metastases can be found; thus, their role in malignant progression needs further clarification [50]. In our study, patients with BRCA1 pathogenic and likely pathogenic variants but not those with BRCA2 variants showed a shorter TTC. Our finding is comparable to the results of Kohli et al., who found that mutations in multiple DNA repair genes (ATM, BRCA1, BRCA2, and CHEK2) were associated with time to ADT failure and survival in metastasized hormone-sensitive PCa [45]. Two AR gene mutations in the ligand binding domain (2105T > A (p. L702H) and 2632A > G (p. T878A)) have been associated with resistance to abiraterone therapy and with a shorter OS in PCa [33]. Mutations in the AR-LBD region are also associated with shorter progression-free survival (PFS) [48]. In our study, the presence of four mutations in the LBD of the AR gene, including the two previously mentioned mutations, tended to be associated with a shorter TTC than observed in patients without mutations in the AR-LBD.

Interestingly, patients with likely pathogenic variants in the MUC16 gene showed a longer OS and a longer time from metastasis to last follow-up. This finding is comparable to the results of Keup et al., who found that three MUC16 variants were associated with a longer time from metastasis to last follow-up in metastatic breast cancer patients [37]. To test whether this is an effect of the large gene size of MUC16, we also analyzed the large-sized gene SYNE1. However, pathogenic and likely pathogenic variants in the SYNE1 gene were not associated with the prognosis of our PCa patients. In gastric cancer, the occurrence of MUC16 gene mutations is correlated with tumor mutational burden and associated with better OS [51]. Recently, Yu et al. showed an association between immune checkpoint inhibitor therapy and survival (OS, PFS) in patients with non-small cell lung cancer with MUC16 variants [52].

When considering all variants, i.e., their functional impact and allele frequency, they were associated with TTC. Group 3, with the highest rate, showed the shortest time for treatment change. A short TTC was correlated with poor OS and DSS in our study (Table 3). Considering that our patient cohort consisted of primarily late-stage progressive disease patients, a short TTC was mainly related to acute therapy failure. However, more studies with larger cohorts are necessary to investigate the sequence of therapies and their effect on gene variants and resistance mechanisms.

In addition, we identified several pathogenic and likely pathogenic gene variants in genes that are of potential relevance for the following existing therapies: (i) DNA repair genes, such as CHEK2, BRCA1/2, ATM, MSH2, MSH6, PALB2, RAD51, ERCC3, MRE11, and NBN; (ii) genes in the PIK3-AKT pathway, such as PIK3CA and PTEN; and (iii) genes in the CDK4/6-RB pathway, such as RB1 and CDKN2A (p16). The occurrence of mutations in DNA repair genes is related to PARP inhibitor therapy, and PTEN deficiency is implicated in AKT-PIK3 pathway blockade, e.g., with ipatasertib in combination with abiraterone [32,34,45,53,54,55,56]. Aberrations in the CDK4/-6-RB1 pathway may suggest application of CDK4/-6 inhibitors [46]. In addition, a clinical activity of pembrolizumab has been shown in metastatic PCa patients with a high microsatellite instability detected in ctDNA [57]. Altogether, our results and previous findings in the literature indicate that characterization of gene variants in cfDNA is relevant for prognosis, therapy prediction, and therapy monitoring.

We also analyzed the occurrence of CTCs and AR-V7-positive CTCs in our PCa patient cohort. We detected CTCs in 29.7% (11/37) of samples and AR-V7-positive CTCs in 10.8% (4/37) of samples. The number of samples with CTCs is expected to be higher in reality, because several PCa patients have undetectable CTCs, despite progressive disease [58], and CTCs with epithelial to mesenchymal transition usually escape CTC detection [13]. In our study cohort, the occurrence of CTCs and AR-V7-positive CTCs was associated with a shorter TTC (*p* = 0.031 and *p* < 0.001). The prognostic role of CTCs in PCa has been clearly shown, as recently reviewed [9]. It has been reported that the prognosis of PCa patients worsens from patients without CTCs to patients with AR-V7-negative CTCs to patients with AR-V7-positive CTCs [59]. Detection of the androgen receptor splice variant AR-V7 has been shown to confer resistance to abiraterone and enzalutamide in PCa [21]. Detection of AR-V7 in CTCs at the RNA and protein levels was associated with shorter PFS and OS after abiraterone or enzalutamide treatment [16]. However, the detection of AR-V7 does not preclude a response to next-generation androgen deprivation therapy [23]. Recently, a study showed that early CTC declines were good predictors of improved outcomes in mCRPC patients treated with docetaxel [60]. Altogether, CTC count has been described as a reliable biomarker for treatment response and prognosis in patients receiving chemotherapy or AR-targeting therapies [61]. Interestingly, in our study, the occurrence of AR-V7-positive CTCs was associated with TP53 point mutations. AR-V7 splicing is regulated by the histone demethylase JMJD1A. JMJD1A has been found to interact with and to promote the recruitment of a member of the heterogeneous nuclear ribonucleoprotein (HNRNP) family, HNRNPF, to the cryptic exon 3b on androgen receptor pre-mRNA for generation of AR-V7 [62]. Another member of the HNRNP family, HNRNPK, is known to function as a cofactor for TP53, e.g., in the DNA damage response [63]. HNRNPK is also well known as a regulator of androgen receptor translation [64]. Recently, mutant TP53 has been shown to have an effect on RNA splicing via the RNA binding protein HNRNPK in pancreatic cancer [65]. Both HNRNPK and HNRNPF are part of the splicing machinery [66]. It is tempting to speculate that a mutated TP53 protein may play a role in the splicing of AR-V7 via HNRNPs.

We found that the level of cfDNA and the occurrence of AR-V7-positive CTCs were correlated and that both were associated with TTC. In addition, the molecular analyses of cfDNA and CTCs also provided complementary information, such as genetic variants and the presence of AR-V7-positive CTCs, which can support therapy decisions. This is in accordance with findings in the literature. As recently reviewed, on the one hand, the assessment of genomic aberrations in cf/ctDNA can potentially predict therapy response and detect mechanisms of resistance, and on the other hand, the presence of CTCs can be a surrogate for disease stage or progression, and can measure therapy response [40,61].

Our study has several limitations. Although the targeted resequencing panel covers 99 genes, more genes are expected to show PCa-relevant gene variants. With the applied CTC detection technique, we were unable to enumerate CTCs or to follow changes in CTC numbers during therapy. In addition to gene variants, splicing variants and epigenetic mechanisms—such as DNA methylation, histone modifications, and expression of noncoding RNAs—can also affect gene or protein expression and may reflect the therapy response, which remained unconsidered in our study. In addition, standardization and the clinical validation of liquid biopsy-based assays to detect predictive biomarkers of resistance is essential.

However, the finding that there are associations of gene variants in cfDNA or CTC occurrence with TTC is of clinical interest. This finding can aid in understanding therapy resistance mechanisms and drive therapeutic decisions in the future, ultimately improving outcomes for PCa patients. Considering that we found variants that are associated with a longer OS and a longer survival time after diagnosis of metastasis (MUC16), it is also conceivable that knowledge of specific variants could lead to less overtreatment or fewer later treatment changes, avoiding stress and new side effects in patients.

## Figures and Tables

**Table 1:** Clinicopathological data, treatment data, and study data.

Parameter	n
Patients	34
Samples	39
Age, median (range) in years	67 (48–89)
Metastasis	
cM0	3
cM1	31
cfDNA, mean (range) in ng/mL	17.93 (2.37–206.25)
CTC-positive patients (samples)	9 (11)
AR-V7-CTC-positive patients (samples)	3 (4)
Survival time, median (range) in months	48 (6–182)
Overall survival	
Alive	24
Dead	10
Disease-specific survival	
Yes	25
No	9
Time from blood sampling to last contact, median (range)	10 (0–61)
Time from metastasis to last follow up, median (range)	31 (11–133)
Time to treatment change	9 (1–40)
Treatment change	
Yes	24
No	8
Not known	2
Pretreatment	
None	6
Depriv	10
AE	8
Tax	3
Multiple	7
Treatment	
AE	10
Tax	18
Chemotherapy/hormone	6
Pretreatment + treatment	
None + chemotherapy/hormone	6
Depriv + AE	5
Tax + AE	4
Mult + AE	2
Depriv + Tax	4
AE + Tax	8
Mult + Tax	5

Abbreviations: None—no pretreatment; Depriv—hormone deprivation; AE—antiandrogens abiraterone/enzalutamide; Tax—taxanes; Mult—multiple (both AE and Tax). Chemotherapy/hormone following None; AE following Depriv; AE following Tax; AE following Mult; Tax following Depriv; Tax following AE; Tax following Mult.

**Table 2:** Overview of cfDNA concentration and detection of CTCs and AR-V7+ CTCs.

Parameter	n
cfDNA, mean (median; range) in ng/mL	17.93 (10.56; 2.37–206.25)
CTC-positive patients (samples)	9 (11)
AR-V7-CTC-positive patients (samples)	3 (4)

**Table 3:** Bivariate correlations between cfDNA, CTCs, and AR-V7-positive CTCs and molecular and prognostic parameters.

Spearman Rho Test		CTC	ARV7-CTC	Time from Blood Sampling to Last Information Collection	Time to Treatment Change	OS	DSS	Variant Groups	TP53 Sequencing Groups
cfDNA	correl. coeff.	0.227	0.346	−0.333	−0.304	0.174	0.109	0.113	0.168
	Sig. (2-side)	0.176	**0.036**	**0.047**	0.071	0.324	0.540	0.505	0.307
CTCs	correl. coeff.		0.535	−0.287	−0.187	0.188	0.236	0.472	0.309
	Sig. (2-side)		**0.001**	0.100	0.289	0.294	0.186	**0.004**	0.063
ARV7-positive CTCs	correl. coeff.			−0.377	−0.378	0.250	0.280	0.219	0.523
	Sig. (2-side)			**0.028**	**0.028**	0.160	0.115	0.206	**0.001**
Time from blood sampling to last information collection	correl. coeff.				0.857	−0.464	−0.441	0.102	−0.246
	Sig. (2-side)				**<0.001**	**0.007**	**0.012**	0.565	0.147
Time to treatment change	correl. coeff.					−0.521	−0.517	0.131	−0.177
	Sig. (2-side)					**0.002**	**0.002**	0.459	0.301
OS	correl. coeff.						0.930	−0.300	0.339
	Sig. (2-side)						**<0.001**	0.095	0.050
DSS	correl. coeff.							−0.345	0.404
	Sig. (2-side)							0.053	**0.018**
Variant groups	correl. coeff.								0.217
	Sig. (2-side)								0.197

Abbreviations: TTC—time to treatment change.

**Table 4:** Kaplan–Meier analysis: association of cfDNA levels, CTCs, or AR-V7-positive CTCs with prognosis.

Parameter	Kaplan–Meier analysis					
	OS		DSS		TTC	
	Months	*p*	Months	*p*	Months	*p*
cfDNA, higher vs. lower levels	42.2 vs. 134.0	**0.014**	42.2 vs. 138.7	**0.004**	6.3 vs. 16.2	**0.001**
CTCs, yes vs. no		n.s.		n.s.	7.6 vs. 15.1	**0.031**
AR-V7-positive CTCs, yes vs. no		n.s.	57.7 vs. 134.8	0.097	4.0 vs. 14.2	**<0.001**

Significant values are in bold. Estimated mean survival is given in months.

**Table 5:** Univariate Cox’s regression analysis: association of cfDNA levels, CTCs, or AR-V7-positive CTCs with prognosis.

Parameter	Univariate Cox’s Regression Analysis					
	OS		DSS		TTC	
	RR (95%CI)	*p*	RR (95%CI)	*p*	RR (95%CI	*p*
cfDNA, higher vs. lower levels	5.53 (1.20–25.31)	**0.027**	7.87 (1.55–40.0453.86)	**0.013**	4.11 (1.57–10.77)	**0.004**
CTCs, yes vs. no		n.s.		n.s.	2.32 (1.02–5.30)	**0.046**
AR-V7-positive CTCs, yes vs. no		n.s.		n.s.	14.75 (3.15–69.1)	**0.001**

Abbreviations: 95% CI—95% confidence interval.

## Data Availability

All data are available in the manuscript and the Supplementary Materials. Detailed datasets used and analyzed during the current study are available from the corresponding author on reasonable request.

## Supplementary Materials

The following data are available online at https://www.mdpi.com/article/10.3390/cells10113223/s1. Table S1: Overview of sequence variants, Table S2: Detection of CTCs and AR-V7+ CTCs with the Adna-Test ProstateCancerPanel AR-V7, Table S3: Cross table: Relationship between TP53 gene variants and CTCs or AR-V7-positive CTCs, Figure S1A: Classification of called variants in cfDNA according to their known functional impact, Figure S1B: Classification of called variants in cfDNA according to variation type, Figure S2: Frequency of pathogenic and likely pathogenic variants per sequence range for the top 15 genes.

## References

[1] Sung H., Ferlay J., Siegel R.L., Laversanne M., Soerjomataram I., Jemal A., Bray F. (2021). Global Cancer Statistics 2020: GLOBOCAN Estimates of Incidence and Mortality Worldwide for 36 Cancers in 185 Countries. CA Cancer J. Clin..

[2] Baca S.C., Prandi D., Lawrence M.S., Mosquera J.M., Romanel A., Drier Y., Park K., Kitabayashi N., MacDonald T.Y., Ghandi M. (2013). Punctuated evolution of prostate cancer genomes. Cell.

[3] Inamura K. (2018). Prostatic cancers: Understanding their molecular pathology and the 2016 WHO classification. Oncotarget.

[4] Lu Y.T., Delijani K., Mecum A., Goldkorn A. (2019). Current status of liquid biopsies for the detection and management of prostate cancer. Cancer Manag. Res..

[5] Doyen J., Alix-Panabières C., Hofman P., Parks S.K., Chamorey E., Naman H., Hannoun-Lévi J.M. (2012). Circulating tumor cells in prostate cancer: A potential surrogate marker of survival. Crit. Rev. Oncol. Hematol..

[6] Armstrong A.J., Eisenberger M.A., Halabi S., Oudard S., Nanus D.M., Petrylak D.P., Sartor A.O., Scher H.I. (2012). Biomarkers in the management and treatment of men with metastatic castration-resistant prostate cancer. Eur. Urol..

[7] Diamond E., Lee G.Y., Akhtar N.H., Kirby B.J., Giannakakou P., Tagawa S.T., Nanus D.M. (2012). Isolation and characterization of circulating tumor cells in prostate cancer. Front. Oncol..

[8] McDaniel A.S., Ferraldeschi R., Krupa R., Landers M., Graf R., Louw J., Jendrisak A., Bales N., Marrinucci D., Zafeiriou Z. (2017). Phenotypic diversity of circulating tumour cells in patients with metastatic castration-resistant prostate cancer. BJU Int..

[9] Pantel K., Hille C., Scher H.I. (2019). Circulating Tumor Cells in Prostate Cancer: From Discovery to Clinical Utility. Clin. Chem..

[10] Mandel P.C., Huland H., Tiebel A., Haese A., Salomon G., Budäus L., Tilki D., Chun F., Heinzer H., Graefen M. (2019). Enumeration and Changes in Circulating Tumor Cells and Their Prognostic Value in Patients Undergoing Cytoreductive Radical Prostatectomy for Oligometastatic Prostate Cancer-Translational Research Results from the Prospective ProMPT trial. Eur. Urol. Focus..

[11] Broncy L., Paterlini-Bréchot P. (2019). Clinical Impact of Circulating Tumor Cells in Patients with Localized Prostate Cancer. Cells.

[12] Josefsson A., Larsson K., Månsson M., Björkman J., Rohlova E., Åhs D., Brisby H., Damber J.E., Welén K. (2018). Circulating tumor cells mirror bone metastatic phenotype in prostate cancer. Oncotarget.

[13] Gorges T.M., Tinhofer I., Drosch M., Röse L., Zollner T.M., Krahn T., von Ahsen O. (2012). Circulating tumour cells escape from EpCAM-based detection due to epithelial-to-mesenchymal transition. BMC Cancer.

[14] Keup C., Storbeck M., Hauch S., Hahn P., Sprenger-Haussels M., Tewes M., Mach P., Hoffmann O., Kimmig R., Kasimir-Bauer S. (2019). Cell-Free DNA Variant Sequencing Using CTC-Depleted Blood for Comprehensive Liquid Biopsy Testing in Metastatic Breast Cancer. Cancers.

[15] Sharp A., Welti J.C., Lambros M.B.K., Dolling D., Rodrigues D.N., Pope L., Aversa C., Figueiredo I., Fraser J., Ahmad Z. (2019). Clinical Utility of Circulating Tumour Cell Androgen Receptor Splice Variant-7 Status in Metastatic Castration-resistant Prostate Cancer. Eur. Urol..

[16] Armstrong A.J., Halabi S., Luo J., Nanus D.M., Giannakakou P., Szmulewitz R.Z., Danila D.C., Healy P., Anand M., Rothwell C.J. (2019). Prospective Multicenter Validation of Androgen Receptor Splice Variant 7 and Hormone Therapy Resistance in High-Risk Castration-Resistant Prostate Cancer: The PROPHECY Study. J. Clin. Oncol..

[17] Josefsson A., Linder A., Flondell Site D., Canesin G., Stiehm A., Anand A., Bjartell A., Damber J.E., Welén K. (2017). Circulating Tumor Cells as a Marker for Progression-free Survival in Metastatic Castration-naïve Prostate Cancer. Prostate.

[18] Scher H.I., Lu D., Schreiber N.A., Louw J., Graf R.P., Vargas H.A., Johnson A., Jendrisak A., Bambury R., Danila D. (2016). Association of AR-V7 on Circulating Tumor Cells as a Treatment-Specific Biomarker With Outcomes and Survival in Castration-Resistant Prostate Cancer. JAMA Oncol..

[19] Josefsson A., Damber J.E., Welén K. (2019). AR-V7 expression in circulating tumor cells as a potential prognostic marker in metastatic hormone-sensitive prostate cancer. Acta Oncol..

[20] Antonarakis E.S., Lu C., Wang H., Luber B., Nakazawa M., Roeser J.C., Chen Y., Mohammad T.A., Chen Y., Fedor H.L. (2014). AR-V7 and resistance to enzalutamide and abiraterone in prostate cancer. N. Engl. J. Med..

[21] Antonarakis E.S., Lu C., Luber B., Wang H., Chen Y., Nakazawa M., Nadal R., Paller C.J., Denmeade S.R., Carducci M.A. (2015). Androgen Receptor Splice Variant 7 and Efficacy of Taxane Chemotherapy in Patients With Metastatic Castration-Resistant Prostate Cancer. JAMA Oncol..

[22] Scher H.I., Graf R.P., Schreiber N.A., Jayaram A., Winquist E., McLaughlin B., Lu D., Fleisher M., Orr S., Lowes L. (2018). Assessment of the Validity of Nuclear-Localized Androgen Receptor Splice Variant 7 in Circulating Tumor Cells as a Predictive Biomarker for Castration-Resistant Prostate Cancer. JAMA Oncol..

[23] Bernemann C., Schnoeller T.J., Luedeke M., Steinestel K., Boegemann M., Schrader A.J., Steinestel J. (2017). Expression of AR-V7 in Circulating Tumour Cells Does Not Preclude Response to Next Generation Androgen Deprivation Therapy in Patients with Castration Resistant Prostate Cancer. Eur. Urol..

[24] Diaz L.A., Bardelli A. (2014). Liquid biopsies: Genotyping circulating tumor DNA. J. Clin. Oncol..

[25] Allen D., Butt A., Cahill D., Wheeler M., Popert R., Swaminathan R. (2004). Role of cell-free plasma DNA as a diagnostic marker for prostate cancer. Ann. N. Y. Acad. Sci..

[26] Altimari A., Grigioni A.D., Benedettini E., Gabusi E., Schiavina R., Martinelli A., Morselli-Labate A.M., Martorana G., Grigioni W.F., Fiorentino M. (2008). Diagnostic role of circulating free plasma DNA detection in patients with localized prostate cancer. Am. J. Clin. Pathol..

[27] Ellinger J., Bastian P.J., Haan K.I., Heukamp L.C., Buettner R., Fimmers R., Mueller S.C., von Ruecker A. (2008). Noncancerous PTGS2 DNA fragments of apoptotic origin in sera of prostate cancer patients qualify as diagnostic and prognostic indicators. Int. J. Cancer.

[28] Feng J., Gang F., Li X., Jin T., Houbao H., Yu C., Guorong L. (2013). Plasma cell-free DNA and its DNA integrity as biomarker to distinguish prostate cancer from benign prostatic hyperplasia in patients with increased serum prostate-specific antigen. Int. Urol. Nephrol..

[29] Wroclawski M.L., Serpa-Neto A., Fonseca F.L., Castro-Neves-Neto O., Pompeo A.S., Machado M.T., Pompeo A.C., del Giglio A. (2013). Cell-free plasma DNA as biochemical biomarker for the diagnosis and follow-up of prostate cancer patients. Tumour. Biol..

[30] Wyatt A.W., Annala M., Aggarwal R., Beja K., Feng F., Youngren J., Foye A., Lloyd P., Nykter M., Beer T.M. (2017). Concordance of Circulating Tumor DNA and Matched Metastatic Tissue Biopsy in Prostate Cancer. J. Natl. Cancer Inst..

[31] Zhang Q., Luo J., Wu S., Si H., Gao C., Xu W., Abdullah S.E., Higgs B.W., Dennis P.A., van der Heijden M.S. (2020). Prognostic and Predictive Impact of Circulating Tumor DNA in Patients with Advanced Cancers Treated with Immune Checkpoint Blockade. Cancer Discov..

[32] González-Billalabeitia E., Conteduca V., Wetterskog D., Jayaram A., Attard G. (2019). Circulating tumor DNA in advanced prostate cancer: Transitioning from discovery to a clinically implemented test. Prostate Cancer Prostatic Dis..

[33] Conteduca V., Wetterskog D., Sharabiani M.T.A., Grande E., Fernandez-Perez M.P., Jayaram A., Salvi S., Castellano D., Romanel A., Lolli C. (2017). Androgen receptor gene status in plasma DNA associates with worse outcome on enzalutamide or abiraterone for castration-resistant prostate cancer: A multi-institution correlative biomarker study. Ann. Oncol..

[34] Goodall J., Mateo J., Yuan W., Mossop H., Porta N., Miranda S., Perez-Lopez R., Dolling D., Robinson D.R., Sandhu S. (2017). Circulating Cell-Free DNA to Guide Prostate Cancer Treatment with PARP Inhibition. Cancer Discov..

[35] Vandekerkhove G., Struss W.J., Annala M., Kallio H.M.L., Khalaf D., Warner E.W., Herberts C., Ritch E., Beja K., Loktionova Y. (2019). Circulating Tumor DNA Abundance and Potential Utility in De Novo Metastatic Prostate Cancer. Eur. Urol..

[36] Butler T.M., Johnson-Camacho K., Peto M., Wang N.J., Macey T.A., Korkola J.E., Koppie T.M., Corless C.L., Gray J.W., Spellman P.T. (2015). Exome Sequencing of Cell-Free DNA from Metastatic Cancer Patients Identifies Clinically Actionable Mutations Distinct from Primary Disease. PLoS ONE..

[37] Keup C., Benyaa K., Hauch S., Sprenger-Haussels M., Tewes M., Mach P., Bittner A.K., Kimmig R., Hahn P., Kasimir-Bauer S. (2020). Targeted deep sequencing revealed variants in cell-free DNA of hormone receptor-positive metastatic breast cancer patients. Cell Mol. Life Sci..

[38] Robinson D., Van Allen E.M., Wu Y.M., Schultz N., Lonigro R.J., Mosquera J.M., Montgomery B., Taplin M.E., Pritchard C.C., Attard G. (2015). Integrative clinical genomics of advanced prostate cancer. Cell.

[39] Taubert H., Meye A., Würl P. (1998). Soft tissue sarcomas and p53 mutations. Mol. Med..

[40] Boerrigter E., Groen L.N., Van Erp N.P., Verhaegh G.W., Schalken J.A. (2020). Clinical utility of emerging biomarkers in prostate cancer liquid biopsies. Expert Rev. Mol. Diagn..

[41] Perkins G., Yap T.A., Pope L., Cassidy A.M., Dukes J.P., Riisnaes R., Massard C., Cassier P.A., Miranda S., Clark J. (2012). Multi-purpose utility of circulating plasma DNA testing in patients with advanced cancers. PLoS ONE.

[42] Zhang Y., Yao Y., Xu Y., Li L., Gong Y., Zhang K., Zhang M., Guan Y., Chang L., Xia X. (2021). Pan-cancer circulating tumor DNA detection in over 10,000 Chinese patients. Nat. Commun..

[43] Fettke H., Kwan E.M., Bukczynska P., Ng N., Nguyen-Dumont T., Southey M.C., Davis I.D., Mant A., Parente P., Pezaro C. (2020). Prognostic Impact of Total Plasma Cell-free DNA Concentration in Androgen Receptor Pathway Inhibitor-treated Metastatic Castration-resistant Prostate Cancer. Eur. Urol. Focus.

[44] Mehra N., Dolling D., Sumanasuriya S., Christova R., Pope L., Carreira S., Seed G., Yuan W., Goodall J., Hall E. (2018). Plasma Cell-free DNA Concentration and Outcomes from Taxane Therapy in Metastatic Castration-resistant Prostate Cancer from Two Phase III Trials (FIRSTANA and PROSELICA). Eur. Urol..

[45] Kohli M., Tan W., Zheng T., Wang A., Montesinos C., Wong C., Du P., Jia S., Yadav S., Horvath L.G. (2020). Clinical and genomic insights into circulating tumor DNA-based alterations across the spectrum of metastatic hormone-sensitive and castrate-resistant prostate cancer. EBioMedicine.

[46] Rodrigues D.N., Boysen G., Sumanasuriya S., Seed G., Marzo A.M., de Bono J. (2017). The molecular underpinnings of prostate cancer: Impacts on management and pathology practice. J. Pathol..

[47] Annala M., Vandekerkhove G., Khalaf D., Taavitsainen S., Beja K., Warner E.W., Sunderland K., Kollmannsberger C., Eigl B.J., Finch D. (2018). Circulating Tumor DNA Genomics Correlate with Resistance to Abiraterone and Enzalutamide in Prostate Cancer. Cancer Discov..

[48] Torquato S., Pallavajjala A., Goldstein A., Toro P.V., Silberstein J.L., Lee J., Nakazawa M., Waters I., Chu D., Shinn D. (2019). Genetic Alterations Detected in Cell-Free DNA Are Associated With Enzalutamide and Abiraterone Resistance in Castration-Resistant Prostate Cancer. JCO Precis. Oncol..

[49] De Laere B., Oeyen S., Mayrhofer M., Whitington T., van Dam P.J., Van Oyen P., Ghysel C., Ampe J., Ost P., Demey W. (2019). TP53 Outperforms Other Androgen Receptor Biomarkers to Predict Abiraterone or Enzalutamide Outcome in Metastatic Castration-Resistant Prostate Cancer. Clin. Cancer Res..

[50] Teroerde M., Nientiedt C., Duensing A., Hohenfellner M., Stenzinger A., Duensing S., Bott S.R.J., Ng K.L. (2021). Revisiting the Role of p53 in Prostate Cancer. Prostate Cancer.

[51] Li X., Pasche B., Zhang W., Chen K. (2018). Association of MUC16 Mutation With Tumor Mutation Load and Outcomes in Patients With Gastric Cancer. JAMA Oncol..

[52] Yu Y., Lin D., Li A., Chen Y., Ou Q., Hu H., Yao H. (2020). Association of Immune Checkpoint Inhibitor Therapy With Survival in Patients With Cancers With MUC16 Variants. JAMA Netw. Open..

[53] Mateo J., Carreira S., Sandhu S., Miranda S., Mossop H., Perez-Lopez R., Nava Rodrigues D., Robinson D., Omlin A., Tunariu N. (2015). DNA-Repair Defects and Olaparib in Metastatic Prostate Cancer. N. Engl. J. Med..

[54] de Bono J.S., De Giorgi U., Rodrigues D.N., Massard C., Bracarda S., Font A., Arranz Arija J.A., Shih K.C., Radavoi G.D., Xu N. (2019). Randomized Phase II Study Evaluating Akt Blockade with Ipatasertib, in Combination with Abiraterone, in Patients with Metastatic Prostate Cancer with and without PTEN Loss. Clin. Cancer Res..

[55] Herberts C., Murtha A.J., Fu S., Wang G., Schönlau E., Xue H., Lin D., Gleave A., Yip S., Angeles A. (2020). Activating AKT1 and PIK3CA Mutations in Metastatic Castration-Resistant Prostate Cancer. Eur. Urol..

[56] Tukachinsky H., Madison R.W., Chung J.H., Gjoerup O., Severson E.A., Dennis L., Fendler B.J., Morley S., Zhong L., Graf R.P. (2021). Genomic analysis of circulating tumor DNA in 3,334 patients with advanced prostate cancer identifies targetable BRCA alterations and AR resistance mechanisms. Clin. Cancer Res..

[57] Barata P., Agarwal N., Nussenzveig R., Gerendash B., Jaeger E., Hatton W., Ledet E., Lewis B., Layton J., Babiker H. (2020). Clinical activity of pembrolizumab in metastatic prostate cancer with microsatellite instability high (MSI-H) detected by circulating tumor DNA. J. Immunother. Cancer.

[58] Sumanasuriya S., Omlin A., Armstrong A., Attard G., Chi K.N., Bevan C.L., Shibakawa A., IJzerman M.J., De Laere B., Lolkema M. (2018). Consensus Statement on Circulating Biomarkers for Advanced Prostate Cancer. Eur. Urol. Oncol..

[59] Antonarakis E.S., Lu C., Luber B., Wang H., Chen Y., Zhu Y., Silberstein J.L., Taylor M.N., Maughan B.L., Denmeade S.R. (2017). Clinical Significance of Androgen Receptor Splice Variant-7 mRNA Detection in Circulating Tumor Cells of Men With Metastatic Castration-Resistant Prostate Cancer Treated With First- and Second-Line Abiraterone and Enzalutamide. J. Clin. Oncol..

[60] Lozano R., Lorente D., Aragon I.M., Romero-Laorden N., Nombela P., Mateo J., Reid A.H.M., Cendón Y., Bianchini D., Llacer C. (2021). Value of Early Circulating Tumor Cells Dynamics to Estimate Docetaxel Benefit in Metastatic Castration-Resistant Prostate Cancer (mCRPC) Patients. Cancers.

[61] Heidrich I., Ačkar L., Mossahebi Mohammadi P., Pantel K. (2021). Liquid biopsies: Potential and challenges. Int J. Cancer.

[62] Fan L., Zhang F., Xu S., Cui X., Hussain A., Fazli L., Gleave M., Dong X., Qi J. (2018). Histone demethylase JMJD1A promotes alternative splicing of AR variant 7 (AR-V7) in prostate cancer cells. Proc. Natl. Acad. Sci. USA.

[63] Wiesmann N., Strozynski J., Beck C., Zimmermann N., Mendler S., Gieringer R., Schmidtmann I., Brieger J. (2017). Knockdown of hnRNPK leads to increased DNA damage after irradiation and reduces survival of tumor cells. Carcinogenesis.

[64] Mukhopadhyay N.K., Kim J., Cinar B., Ramachandran A., Hager M.H., Di Vizio D., Adam R.M., Rubin M.A., Raychaudhuri P., De Benedetti A. (2009). Heterogeneous nuclear ribonucleoprotein K is a novel regulator of androgen receptor translation. Cancer Res..

[65] Escobar-Hoyos L.F., Penson A., Kannan R., Cho H., Pan C.H., Singh R.K., Apken L.H., Hobbs G.A., Luo R., Lecomte N. (2020). Altered RNA Splicing by Mutant p53 Activates Oncogenic RAS Signaling in Pancreatic Cancer. Cancer Cell.

[66] Zhang Y., Qian J., Gu C., Yang Y. (2021). Alternative splicing and cancer: A systematic review. Signal. Transduct Target. Ther..

