# Supplementary material for: Cell-Free DNA Variant Sequencing Using Plasma and AR-V7 Testing of Circulating Tumor Cells in Prostate Cancer Patients
Source: Cells. 2021 Nov 18;10(11):3223. doi: 10.3390/cells10113223 (PMC8620951; doi:10.3390/cells10113223)
Supplement: Supplementary file 1 [file cells-10-03223-s001.zip › cells-1446836-Suppl. Table 2 and 3.pdf]

Supplemental Tables 2 and 3

Cell-free DNA variant sequencing using plasma and AR-V7 testing of CTCs in prostate cancer patients

Verena Lieb[1,2], Amer Abdulrahman[1,2], Katrin Weigelt[1,2], Siegfried Hauch[3], Michael Gombert[3], Juan Guzman[1,2], Laura Bellut[1,2], Peter J. Goebell[1,2], Robert Stöhr[2,4], Arndt Hartmann[2,4], Bernd Wullich[1,2], Helge Taubert[1,2,†,*] and Sven Wach[1,2,†]

Table S2: Detection of CTCs and AR-V7+ CTCs with the Adna-Test ProstateCancerPanel AR-V7

Patient-No.	CTC_No.	CTC	AR-V7	Seq. sample	CTC_No.	CTC	AR-V7	Seq. sample	CTC_No.	CTC	AR-V7	Seq. sample
1	CTC-30	negative	negative	1								
2	CTC-46	negative	negative	2								
3	CTC-49	negative	negative	3								
4	CTC-54	**positive**	**positive**	4								
5	CTC-62	negative	negative	5								
6	CTC-63	**positive**	**positive**	6	CTC-84	**positive**	**positive**	12	CTC-14	n.d.	n.d.	29
7	CTC-74	**positive**	negative	7								
8	CTC-75	**positive**	**positive**	8								
9	CTC-77	negative	negative	9								
10	CTC-79	negative	negative	10								
11	CTC-83	**positive**	negative	11								
12	CTC-89	**positive**	negative	13	CTC-105	negative	negative	21				
13	CTC-90	negative	negative	14								
14	CTC-91	negative	negative	15								
15	CTC-92	negative	negative	16								
16	CTC-95	negative	negative	17								

17	CTC-96	**positive**	negative	18				
18	CTC-99	negative	negative	19	CTC-118	negative	negative	24
19	CTC-102	negative	negative	20				
20	CTC-110	negative	negative	22				
21	CTC-115	negative	negative	23				
22	CTC-127	negative	negative	25				
23	CTC-131	negative	negative	26				
24	CTC-133	**positive**	negative	27				
25	CTC-143	negative	negative	28				
26	CTC-25	n.d.	n.d.	30				
27	CTC-129	negative	negative	31				
28	CTC-137	negative	negative	32				
29	CTC-157	negative	negative	33				
30	CTC-168	**positive**	negative	34	CTC-191	**positive**	negative	38
31	CTC-173	negative	negative	35				
32	CTC-178	negative	negative	36				
33	CTC-180	negative	negative	37				
34	CTC-204	negative	negative	39				

Abbreviation: Seq. Sample, sequenced sample
Detection of CTCs or AR-V7-positive CTCs is marked in bold

cells MDPI

Table S3 Cross table: Relationship between TP53 gene variants and CTCs or AR-V7-positive CTCs

Cross table			CTCs		ARV7-positive CTCs		Total
			no	yes	no	yes	
TP53 gene variants (pathogenic and likely pathogenic)	Group 1: without gene variants	n	16	4	20	0	20
	Group 2: with frameshift mutations	n	7	2	9	0	9
	Group 3: with point mutations	n	3	5	4	4	8
Total		n	26	11	33	4	37
