# Supplementary material for: Cell-Free DNA Variant Sequencing Using Plasma and AR-V7 Testing of Circulating Tumor Cells in Prostate Cancer Patients
Source: Cells. 2021 Nov 18;10(11):3223. doi: 10.3390/cells10113223 (PMC8620951; doi:10.3390/cells10113223)
Supplement: Supplementary file 1 [file cells-10-03223-s001.zip › Supplemental Figures S1 and S2.pdf]

Supplemental Figures S1 and S2

# Cell-free DNA variant sequencing using plasma and AR-V7 testing of CTCs in prostate cancer patients

**Verena Lieb[1,2], Amer Abdulrahman[1,2], Katrin Weigelt[1,2], Siegfried Hauch[3], Michael Gombert[3], Juan Guzman[1,2], Laura Bellut[1,2], Peter J. Goebell[1,2], Robert Stöhr[2,4], Arndt Hartmann[2,4], Bernd Wullich[1,2], Helge Taubert[1,2,†,*] and Sven Wach[1,2,†]**

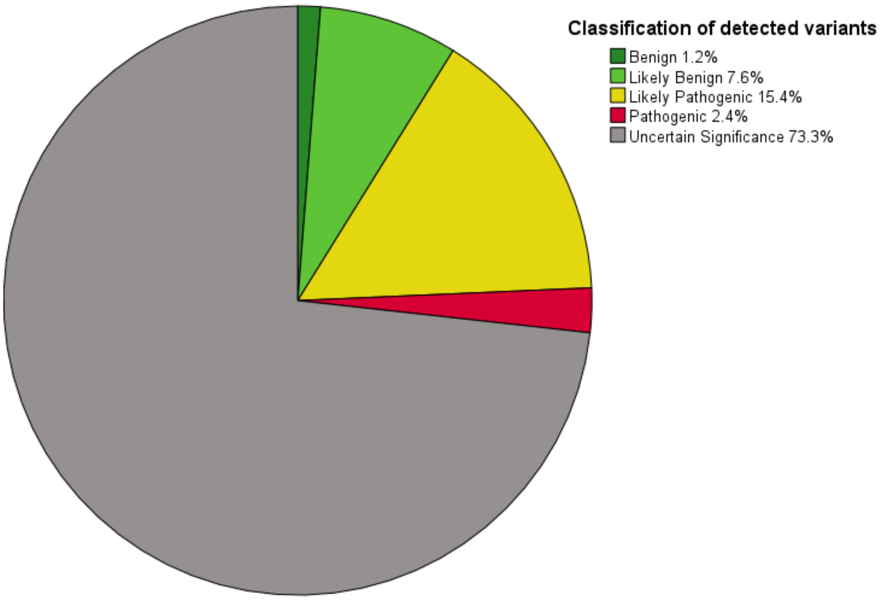


Figure S1A: Classification of called variants in cfDNA according to their known functional impact

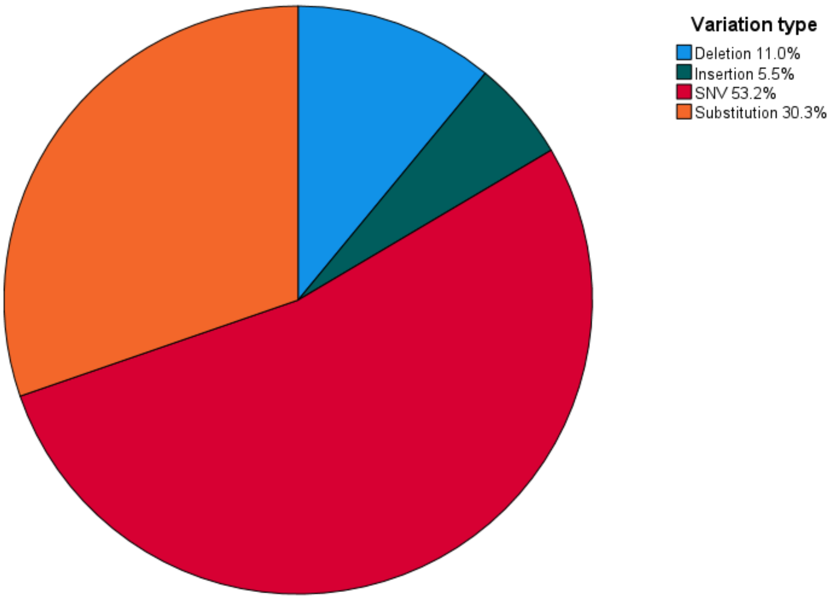


Figure S1B: Classification of called variants in cfDNA according to variation type

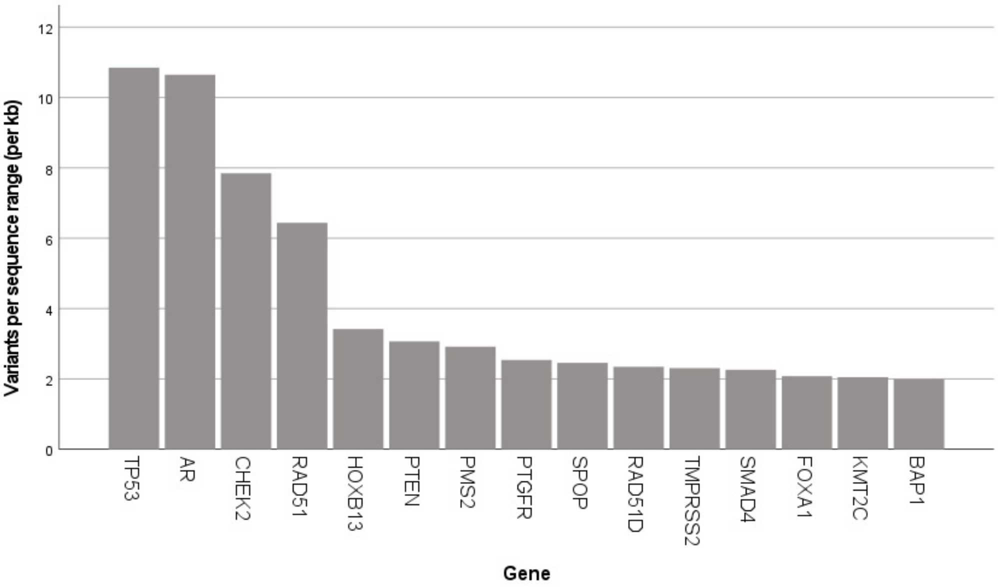


Figure S2: Frequency of pathogenic and likely pathogenic variants per sequence range for the top 15 genes
